# Towards an International Consensus on the Prevention, Treatment, and Management of High-Risk Substance Use and Overdose among Youth

**DOI:** 10.3390/medicina58040539

**Published:** 2022-04-13

**Authors:** Michael Krausz, Jean N. Westenberg, Vivian Tsang, Janet Suen, Martha J. Ignaszewski, Nickie Mathew, Pouya Azar, Maurice Cabanis, Julie Elsner, Marc Vogel, Renske Spijkerman, Laura Orsolini, Dzung Vo, Eva Moore, Jessica Moe, Johannes Strasser, Patrick Köck, Calin Marian, Kenneth M. Dürsteler, Markus Backmund, Jeanette Röhrig, Marianne Post, Hans Haltmayer, Wolfgang Wladika, Thomas Trabi, Christian Muller, Gerhard Rechberger, Maree Teesson, Michael Farrell, Grant Christie, Sally Merry, Mostafa Mamdouh, Rachel Alinsky, Sharon Levy, Marc Fishman, Richard Rosenthal, Kerry Jang, Fiona Choi

**Affiliations:** 1 Department of Psychiatry, Faculty of Medicine, University of British Columbia, Vancouver, BC V6T 2A1, Canada; m.krausz@mac.com (M.K.); vivianwltsang@alumni.ubc.ca (V.T.); jsuen@cheos.ubc.ca (J.S.); martha.ignaszewski@cw.bc.ca (M.J.I.); nickmathew@gmail.com (N.M.); pouya1844@gmail.com (P.A.); mostafamamdouh17@gmail.com (M.M.); kerryjang@shaw.ca (K.J.); fiona.choi@ubc.ca (F.C.); 2 Center for Mental Health, Clinic for Addiction Medicine and Addictive Behavior, Klinikum Stuttgart, 70374 Stuttgart, Germany; maurice.cabanis@gmail.com (M.C.); jeanette.roehrig@gmail.com (J.R.); 3 Complex Pain and Addiction Service, Vancouver General Hospital, Vancouver, BC V5Z 1M9, Canada; 4 BC Children’s Hospital, Vancouver, BC V6H 3N1, Canada; dvo@cw.bc.ca; 5 BC Mental Health & Substance Use Services, Provincial Health Services Authority, Vancouver, BC V5Z 3L7, Canada; 6 Department of Psychiatry, Psychiatric University Clinics Basel, University of Basel, 4002 Basel, Switzerland; j.elsner@fingon.net (J.E.); marc.vogel@upkbs.ch (M.V.); hannes.strasser@upkbs.ch (J.S.); patrick.koeck@upk.ch (P.K.); calin.marian@upk.ch (C.M.); kenneth.duersteler@upkbs.ch (K.M.D.); 7 Psychiatric Services of Thurgovia, Division of Substance Use Disorders, 8596 Münsterlingen, Switzerland; 8 Parnassia Addiction Research Centre (PARC), Brijder Addiction Treatment, Parnassia Group, 2512 The Hague, The Netherlands; renske.spijkerman@brijder.nl (R.S.); mariannepost@hotmail.com (M.P.); 9 Unit of Clinical Psychiatry, Department of Neurosciences/DIMSC, School of Medicine and Surgery, Polytechnic University of Marche, 60121 Ancona, Italy; laura.orsolini@ospedaliriuniti.marche.it; 10 Psychopharmacology, Drug Misuse and Novel Psychoactive Substances Research Unit, School of Life and Medical Sciences, University of Hertfordshire, Hatfield AL10 9EU, UK; 11 Division of Adolescent Health and Medicine, Department of Pediatrics, Faculty of Medicine, University of British Columbia, Vancouver, BC V5Z 1M9, Canada; eva.moore@cw.bc.ca; 12 Department of Emergency Medicine, Faculty of Medicine, University of British Columbia, Vancouver, BC V5Z 1M9, Canada; jessica.moe@gmail.com; 13 BC Centre for Disease Control, Vancouver, BC V5Z 4R4, Canada; 14 Praxiszentrum im Tal, 80331 Munich, Germany; markus.backmund@p-i-t.info; 15 Ludwig-Maximilians-University, 80539 Munich, Germany; 16 Suchthilfe Wien, gGmbH, 1060 Vienna, Austria; hans.haltmayer@suchthilfe.at; 17 Department of Neurology and Psychiatry of Childhood and Adolescence, Klinikum Klagenfurt am Wörthersee, 9020 Klagenfurt, Austria; wolfgang.wladika@kabeg.at; 18 Department for Child and Adolescent Psychiatry and Pschotherapy, LKH Graz II, 8053 Graz, Austria; thomas.trabi@lsf-graz.at; 19 Department of Child & Youth Psychiatry and Psychotherapy, Psychosocial Service Burgenland GmbH, 7000 Eisenstadt, Austria; praxis-cm@gmx.at; 20 Verein Dialog, Integrative Suchtberatung Gudrunstraße, 1100 Wien, Austria; gerhard.rechberger@dialog-on.at; 21 The Matilda Centre for Research in Mental Health and Substance Use, The University of Sydney, Sydney, NSW 2006, Australia; maree.teesson@sydney.edu.au; 22 National Drug and Alcohol Research Centre, University of New South Wales, Sydney, NSW 2031, Australia; michael.farrell@unsw.edu.au; 23 Department of Psychological Medicine, Faculty of Medical and Health Sciences, University of Auckland, Auckland 1023, New Zealand; g.christie@auckland.ac.nz (G.C.); s.merry@auckland.ac.nz (S.M.); 24 Department of Neuropsychiatry, Tanta University, Tanta 31527, Egypt; 25 Division of Adolescent/Young Adult Medicine, Johns Hopkins University School of Medicine, Baltimore, MD 21205, USA; ralinsk1@jhmi.edu; 26 Adolescent Substance Use and Addiction Program, Boston Children’s Hospital, Boston, MA 02115, USA; sharon.levy@childrens.harvard.edu; 27 Department of Psychiatry and Behavioral Sciences, Johns Hopkins University, Baltimore, MD 21205, USA; mjfishman@comcast.net; 28 Mountain Manor Treatment Center, Baltimore, MD 21229, USA; 29 Department of Psychiatry and Behavioral Health, Stony Brook University, New York, NY 11794, USA; richard.rosenthal@stonybrookmedicine.edu

**Keywords:** adolescence, consensus, delphi study, high-risk substance use, substance use disorder, overdose, youth

## Abstract

*Background and Objectives*: Now more than ever, there is an obvious need to reduce the overall burden of disease and risk of premature mortality that are associated with mental health and substance use disorders among young people. However, the current state of research and evidence-based clinical care for high-risk substance use among youth is fragmented and scarce. The objective of the study is to establish consensus for the prevention, treatment, and management of high-risk substance use and overdose among youth (10 to 24 years old). *Materials and Methods*: A modified Delphi technique was used based on the combination of scientific evidence and clinical experience of a group of 31 experts representing 10 countries. A semi-structured questionnaire with five domains (clinical risks, target populations, intervention goals, intervention strategies, and settings/expertise) was shared with the panelists. Based on their responses, statements were developed, which were subsequently revised and finalized through three iterations of feedback. *Results:* Among the five major domains, 60 statements reached consensus. Importantly, experts agreed that screening in primary care and other clinical settings is recommended for all youth, and that the objectives of treating youth with high-risk substance use are to reduce harm and mortality while promoting resilience and healthy development. For all substance use disorders, evidence-based interventions should be available and should be used according to the needs and preferences of the patient. Involuntary admission was the only topic that did not reach consensus, mainly due to its ethical implications and resulting lack of comparable evidence. *Conclusions:* High-risk substance use and overdoses among youth have become a major challenge. The system’s response has been insufficient and needs substantial change. Internationally devised consensus statements provide a first step in system improvement and reform.

## 1. Introduction

Now more than ever, there is an obvious need to reduce the overall burden of disease and risk of premature mortality that are associated with mental health and substance use disorders among young people [1]. Two of the most common reasons for mortality among adolescents and young adults in high-income countries are overdose and suicide [2,3,4,5,6,7,8]. In the context of the overdose crisis, noticeable changes in life expectancy have been reported in Canada and the United States, which are largely attributable to drug overdoses among young individuals [9,10]. Moreover, although Europe is not currently experiencing a similar overdose crisis, the increasingly potent drug market in North America should raise the alarm globally, including in European countries [11,12,13]. In recent years, there has been a significant rise in the number of novel psychoactive substances (NPS) reported by the EU Early Warning System, including synthetic opioids such as fentanyl and carfentanil, but also synthetic cannabinoids, designer benzodiazepines, and stimulants, which pose a significant risk to vulnerable individuals who use substances in Europe, especially youth [14,15,16]. Finally, the COVID-19 pandemic has further increased the risk of developing or worsening mental illness and substance use disorders among adolescents due to uncertainties regarding academics, careers, social life, and other general concerns [17,18].

However, youth are less able to access treatment for substance use disorder (SUD) compared to adults, and the treatment options offered to youth are substantially different and limited compared to those offered to adults [19]. Among youth who experienced a nonfatal opioid overdose, only 2% received pharmacotherapy within 30 days of overdosing and 29% received behavioral health services alone; more than 70% received no addiction treatment [20]. Behavioural interventions without medication are the mainstay for treatment of SUD among youth, despite guidelines recommending the use of pharmacotherapy in young patients [21,22,23]. For example, clinical trial data and several observational studies broadly support the use of medications such as buprenorphine, methadone, and naltrexone for opioid use disorder (OUD), which are associated with reduced overdose and opioid-related morbidity when compared with other treatment pathways such as no treatment and behavioral interventions alone [24,25,26]. However, while some guidelines recommend the use of pharmacotherapy and clinical trial data has demonstrated positive outcomes, regulatory boards in different countries have established different policies. For example, in the US, the only Food and Drug Administration (FDA)-approved medications for adolescent OUD are buprenorphine (only for those aged 16 or older) and methadone (only for those aged 18 or younger who have failed two prior 28-day rehab treatments). For other SUDs, pharmacotherapies used in adults, such as naltrexone, acamprosate, and disulfiram for alcohol use disorder, and varenicline, bupropion, and nicotine replacement therapy for nicotine use disorder, have not yet been approved for individuals under 18 years of age, limiting the treatment response available and leading to elevated substance-related harm and mortality in youth with SUDs [27,28,29,30]. For illicit SUDs (e.g., stimulant use disorder, amphetamine use disorder), which have no FDA-approved medications for adults or adolescents, future research must also include young patients to evaluate the safety and efficacy of pharmacotherapy options in this vulnerable age group [29]. In Europe, the use of evidence-based treatments for youth, such as OAT, appears to be more widespread than in North America and generally approved by regulatory boards in specific countries (e.g., Austria [31,32]), but drug markets are dynamic in nature and must be met with proactive responses that stay ahead of an already escalating problem and potential NPS crisis [13,33,34,35,36].

Although there exist national resources offering pediatric-focused training and support, as well as a wealth of policy statements, clinical reports, and educational materials, there still seems to be a paucity of pediatric addiction medicine subspecialists [30,37]. There are few youth-focused providers (i.e., child and adolescent psychiatrists, pediatricians, and adolescent medicine physicians) who are able to prescribe pharmacotherapy for SUDs, and fewer who are willing to, or have experience, prescribing other medications shown to improve outcomes for SUDs off-label [30,38]. More generally, there is a dearth of substance use-trained youth-focused physicians [39]. Physicians’ lack of confidence in prescribing for SUDs, in addition to the unfamiliarity and stigma surrounding adolescent substance use, has led to the limited use of a wide range of evidence-based treatment options [40,41]. For example, many providers view opioid agonist treatment (OAT) as a ‘last resort’ for youth with OUD, often waiting until youth have relapsed or until they have experienced severe adverse consequences from other forms of treatment [41,42,43]. All of this is aggravated across countries by the arbitrary age barrier of 18 for the transition from child and adolescent to adult psychiatry, which limits the development of competences addressing the similar needs of the population aged 18 to 25 years. If the treatment offered is not engaging and retention is low, young patients are often blamed for a lack of motivation. Essential harm reduction services that are proven to reduce mortality also rarely accommodate the needs of adolescents and are seldom used by youth [44,45].

The lack of comprehensive youth-focused treatment approaches represents a failure to address the mental health and substance use needs of adolescents and young adults, especially now in the face of unprecedented morbidity and mortality. Prioritizing prevention and early intervention and promoting access to appropriate treatment resources are the best strategies to reduce overdose, as is performed in parts of Europe, such as Switzerland and Austria [31,46]. Specifically, in Switzerland in the early 90s, the successful change from a repressive attitude against street-entrenched drug-using youth to an active policy of supportive measures to prevent health and social deterioration was facilitated by coalition building and knowledge brokering [47]. The key to the paradigm shift in Swiss drug policy was the building of networks to develop evidence-based solutions and the mapping out of scientific consensus and controversies in order to create a framework of best standard practices, provide a clear baseline for future improvements, and define research agendas and immediate priorities [47].

With this in mind, the main objective of this study is to establish consensus for the screening, treatment, and management of high-risk substance use and overdose among adolescents and young adults through a transparent Delphi process. This exercise is not to be perceived as an attempt to establish treatment guidelines, but rather as a concerted effort by an international community of practice to achieve clarity and agree on certain practices. This consensus statement is therefore intended for global, regional, national, and local stakeholders (e.g., clinicians, nurses, allied health professionals, researchers, health policy administration) in securing a healthy future for youth and improving the consistency of substance use care for adolescents and young adults. For the purposes of this document, high-risk substance use is defined as any use of substances with a high-risk of adverse outcomes and includes misuse of prescription drugs, use of illicit drugs, and use of injection drugs [48]. Due to the rising trends and an urgent current need, the focus of this consensus statement is on substances of high-risk and overdose, rather than all substance use risk. Moreover, the term ‘youth’ is defined as the developmental period that begins with changes in puberty and culminates in the assumption of adult roles, generally defined as being between 10 and 24 years of age and encompassing both adolescents and young adults.

## 2. Materials and Methods

### 2.1. Panel Selection

An international group of 35 experts were invited to participate in the consensus process based on clinical and academic expertise in the field of youth substance use disorders (SUD). In selecting participants to invite, a number of factors were considered, including previous contributions to and standing in the fields of addiction psychiatry and adolescent medicine, recognized leaders in respective countries, while avoiding over-representation of a single country, and attempting to ensure ‘disciplinary’ diversity. Achieving a balance meant that not everyone who has contributed significantly to the field could be invited, hence the title of ‘towards a consensus’ as the authors make no claims to have achieved a universal consensus. A similar approach has been used in similar consensus building projects [49]. No one approached refused to take part, but some were unable to participate in any of the steps of the Delphi process and therefore could not take part in the project. In total, 31 participated in the Delphi process, hereafter referred to as “panelists” (Table 1).

### 2.2. Delphi Process

**Figure 1:**
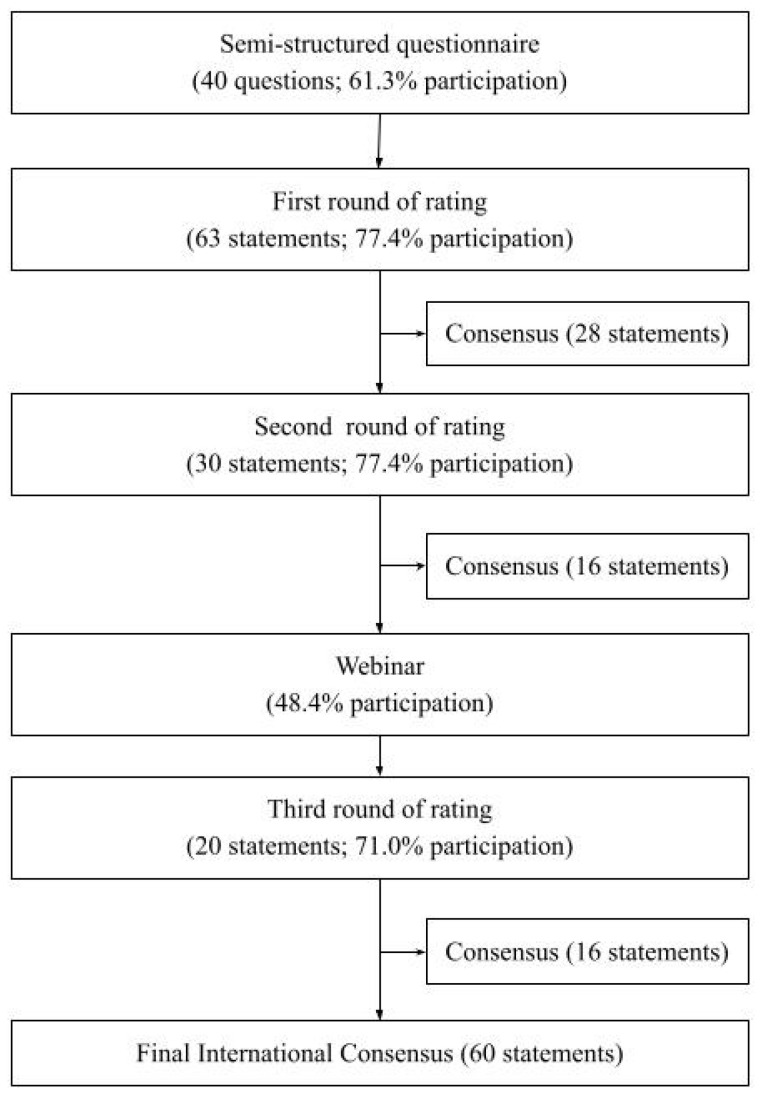
Flowchart of Delphi Process.

A modified Delphi process was conducted according to the steps described below (Figure 1). Generally, the Delphi method typically involves administering questionnaires to expert participants in an iterative series of rounds. The Delphi method is a recognised and well-established technique for reaching consensus that makes the best use of available information, be that scientific data or the collective wisdom of the participants [49,50,51].

1. A semi-structured questionnaire was constructed and organized into the following five domains with corresponding actionable goals: (a) outlining clinical risk, (b) determining target populations, (c) defining intervention goals, (d) recognizing evidence-based intervention strategies, (e) identifying appropriate treatment setting and expertise. The domains were identified through a non-systematic review of the literature and a published narrative literature review on treatment approaches for youth with high-risk opioid use and OUD [25]. Though the narrative literature review focused solely on opioid use, this Delphi study was more comprehensive due to the diversity of the expert panel and the different trends in substance use and overdose internationally. The final version of the semi-structured questionnaire included 40 questions (Supplementary Materials S1), which was sent to the international panel;
2. Based on the responses collected from the semi-structured questionnaire, 63 initial statements were generated. These initial statements were designed by the research group (JNW, VT, JS, KJ, FC) and were meant to encapsulate the responses provided by the panelists. In some instances, statements were created from panelists’ responses verbatim, while others were slightly modified to ensure that the spirit of all responses was coherent with the statement;
3. All statements were sent to the panelists who were asked to rate all statements on a scale of 1–5 (1 = strongly disagree, 2 = somewhat disagree, 3 = neutral, 4 = somewhat agree, 5 = strongly agree) based on their knowledge and clinical experience. If panelists disagreed with a statement, they were given the opportunity to provide comments on the content and/or the phrasing of the statement. Panelists were also able to propose additional statements;
4. After all ratings were received, consensus was calculated. Consensus for each statement was defined as at least 95% of all ratings being greater than or equal to three (“strongly agree”, “somewhat agree”, and “neutral”), a procedure that has been used in recent consensus statements [49,50]. All statements without consensus were revised by the research group (JNW, VT, JS, KJ, FC) based on panelists’ feedback and sent out for a second round of rating, along with any additional statements proposed by the panelists;
5. Consensus was calculated for this subgroup of statements using the same a priori defined rules. Statements that still did not reach expert consensus were deemed controversial and were discussed with the panelists during an online webinar;
6. The purpose of the webinar was to have clear and robust direct verbal discussion that allowed disagreements to be aired and mutually understood and that facilitated a sense of the group having a clearly defined and shared goal [49]. The webinar was organized as follows: summary of results, discussion of identified areas of disagreement, revision of statements that did not reach consensus, and discussion on next steps and priorities. All statements discussed in the webinar were revised by the research group (JNW, VT, JS, KJ, FC) based on the comments made by the panelists and sent out for a third round of rating;
7. Consensus was calculated for this subgroup of statements using the same a priori defined rules. The full text of the international consensus statement was then prepared by the research group (JNW, VT, JS, KJ, FC) and lead author (MK), and shared with the Delphi panel, with a final opportunity to comment on the text. Based on the final round of feedback and comments, the international consensus statement document was finalized.

An international consensus was developed after a narrative review was used to create a semi-structured questionnaire, which was distributed to a panel of 31 experts. Responses to the semi-structured questionnaire were used to create initial statements. The statements then underwent 3 rounds of rating to reach consensus. For each step, the percent participation is shown, along with the number of initial questions or statements presented to the panelists in that step. The number of statements that reached consensus after each round of rating is also shown.

### 2.3. Ethics

This study received approval from the University of British Columbia’s Behavioural Research Ethics Board (H20-03464). Responses were collected electronically through a UBC-hosted version of Qualtrics. Responses to the open-ended questionnaire and to the rating questionnaires were kept anonymous between the experts but could not be kept anonymous during the online webinar, nor to the research group (JNW, VT, JS, KJ, FC) so that non-respondents could be pursued.

## 3. Results

### 3.1. Semi-Structure Questionnaire

The semi-structured questionnaire asked each panelist to share their views on the principles of prevention, treatment, and management of high-risk substance use in youth (Supplementary Materials S1). Of the 31 panelists, 19 responded to the semi-structured questionnaire (61.3%). A total of 63 initial statements were generated from responses, which provided the structure for the first round of rating.

### 3.2. First Round of Rating

Based on the 63 initial statements, a questionnaire was developed using an online survey platform (Qualtrics). Of the 31 panelists, 24 participated in the first round of ratings (77.4%). Roughly half of the statements reached consensus (28; 44.4%). Of the remaining 35 statements, 18 were revised for another round of rating, while 17 were removed due to redundancy or repetition. In addition, 14 new statements were created based on suggestions provided by the panelists. The 18 revised statements and the 14 new statements provided the structure for the second round of rating.

### 3.3. Second Round of Rating

Of the 31 panelists, 24 participated in the second round of ratings (77.4%). Of the 32 statements distributed, half reached consensus (16; 50.0%), while 14 were revised and two were removed; six new statements were created. The 14 revised statements and six new statements were used to guide the discussions in the webinar.

### 3.4. Webinar

Fifteen panelists attended the online webinar (48.4%). The following five major topics were presented to the panelists that had been controversial in the previous rounds of rating: mental health and substance use screening (two statements), goals of treatment (two statements), involuntary admission (one statement), first-line treatments (five statements), and family involvement (three statements). Based on suggestions made by panelists in the webinar, the 20 statements were revised for the third round of rating.

### 3.5. Third Round of Rating

Of the 31 panelists, 22 participated in the third round of ratings (71.0%). Of the 20 statements distributed, 16 reached consensus and four were removed. Of note, the statement on involuntary admission, which had been a major topic of discussion in the webinar, did not reach consensus in the third round.

### 3.6. Final Consensus

In summary, consensus was reached on 28 of the 63 statements in the first round of rating, 16 of the 32 statements in the second round of rating, and 16 of the 20 in the third round of rating, resulting in consensus for 60 statements (Table 2). Among the 60 statements, some have already reached consensus in the medical community but have been included nevertheless to underline their importance.

Of the 31 panelists, 17 participated in all three rounds of rating, four participated in two of the three rounds, and nine participated in one of the three rounds. Only one panelist did not participate in a round of rating but responded to the semi-structured questionnaire.

## 4. Discussion

Globally, youth-tailored services for high-risk substance use are rare. Many treatment paradigms for high-risk substance use among youth are focused on abstinence without considering other goals or options. This is unlike treatment protocols for adults and is not based on clinical research evidence [21,22,42,52,53,54]. Though principles of care have been recently developed, there is still a pressing need to bridge the gap in clinical practice and treatment guidance [55]. A necessary first step in addressing reform and system development is by defining common ground, acknowledging differences in opinions, and supporting evidence-based decision-making. A consensus statement for high-risk substance use among youth was therefore developed with the intention of providing clinicians, allied health professionals, and healthcare systems internationally with a framework to work from and to better integrate adolescent and young adult substance use treatment with general medical care. The next step would be the design of specific care recommendations or formal guidelines, and the support and implementation of evidence-based solutions according to the regional context of each healthcare system.

Through the consensus process, the panel placed emphasis on a few important points. To reduce high-risk substance use, SUD prevalence, and overdose in young patients, evidence-based practices need to be implemented and integrated into primary care. A continuum of care is needed, from lifestyle mentoring, harm reduction, and targeted prevention to early crisis response, with a clinical trajectory addressing all major developmental challenges. Services and resources (e.g., welfare, perinatal, harm reduction) must be tailored to the specific therapeutic needs of vulnerable youth. In previous studies, street-involved young people have reported inadequate social support and abstinence-focused treatment methods as impediments to reducing or stopping injection drug use, whereas appropriate access to harm reduction-informed youth-focused services and provision of housing and social support increased refraining [44,56]. The family unit is also a major resource in an adolescent’s treatment process, but parental/family involvement in the management and treatment of SUD in youth can be complicated. Family therapy, along with culturally syntonic family-based prevention programs, has been found to be effective for SUD treatment but should not be a barrier to care [57,58,59,60,61]. In the case of serious life-threatening events and emergency situations such as nonfatal overdoses or suicide attempts, a youth-appropriate crisis response system needs to be established, which is far from the current standard of care [20,62]. This should include a diverse range of available and effective treatment options and harm reduction approaches such as naloxone distribution, pharmacotherapy, counselling, case management, and family involvement. Due to the critical nature of OUD, effective medications for adults should be made available to youth, and exceptions to certain medications should only be made if clinical research finds them ineffective. Without more clinical research and health system improvement, the effective prevention, treatment, and management of high-risk substance use among youth is nearly impossible.

Despite agreement among the experts, there were some areas of contention. Screening of all youth for mental health problems, substance use problems, and related risks was called into question from a feasibility standpoint due to the many illnesses already screened for in primary healthcare. However, a broader evaluation of the healthcare system as a whole is needed if screening for mental and substance use disorders, the leading causes of disability in children and youth, is unfeasible [1]. To minimize the workload among primary care and mental health providers, the use of standardized youth-specific tools such as the CRAFFT assessment tool, Substances and Choice Scale (SACS), and the Measurements in Addiction for Triage and Evaluation for Young People (MATE-Y), which are validated and easy to use, should be more broadly used in primary care [63,64,65]. Online platforms that provide risk assessment, psychoeducation, and targeted intervention are being developed, with the potential to reduce harm and address the substantial burden of disease attributed to substance use among youth [66,67,68]. Moreover, the only statement with no consensus concerned involuntary admission, a complex ethical issue in youth and still highly contentious in adults. Although most experts agreed that involuntary admission could be considered as part of a comprehensive treatment approach in extremely complex and high-risk situations, most experts also drew attention to the potential consequences it could have on the trust and engagement of patients, as well as the importance of personalized care and often the need for case-by-case considerations. Recent literature has demonstrated that youth who are hospitalized involuntarily report more distrust, including perceiving inpatient treatment to be more punitive than therapeutic [69,70]. A clear distinction should also be drawn between involuntary admission and involuntary treatment, the former of which is intended to interrupt a cycle with stabilization care. Nevertheless, the panel did not come to a consensus on an approach of this much weight and magnitude without first gathering scientific evidence. Research is critical to quantifying the risks and benefits of this approach and evaluating the effects of this approach on the long-term health outcomes of youth. In reality, involuntary treatment is often used in a crisis that has escalated due to mostly inappropriate treatments that came too late, paired with gaps in the continuum of care. This discussion should focus on how to intervene and engage the patients early. The innovative work performed with young patients experiencing first-episode psychosis demonstrates the benefit of early intervention approaches, which contributed to a paradigm shift in the system of care with better health outcomes [71,72]. A similar approach is warranted for traumatized youth with high-risk substance use, who have some of the highest rates of mortality among young mental health patients [2,8,73].

The agreed-upon statements developed by the international expert panel are consistent with other existing guidelines from organizations such as the American Academy of Pediatrics, Society for Adolescent Health and Medicine, American Society of Addiction Medicine, BC Centre for Substance Use, New Zealand Minister of Health, Australian Government Department of Health, and Austrian Society for Child and Adolescent Psychiatry, and should not be a replacement [21,22,23,31,74,75]. We stress that our primary goal is to present a consensus framework developed by a large group of experts from different countries and different cultures and to provide direction to a dysfunctional system towards a more structured and comprehensive approach, to be reflected in future guidelines and healthcare system development. This consensus statement can be viewed as a step towards fully engaging the wider community of clinicians, nurses, and allied healthcare professionals through education, specialized training in addiction and psychiatric care, and promoting awareness within institutions. With this international consensus, we encourage discussions that can be used to identify points of agreement and contestation, ultimately driving the conversation forward. Though it would be difficult to address all 60 consensus statements individually, each of the five domains can provide the groundwork for more specific research and clinical activities (e.g., diversifying evidence-based intervention strategies, adding vulnerable target populations such as boys and young men, girls and young women, black/African American youth, Indigenous youth, Hispanic/Latino youth, LGBTQ+ youth, justice-involved youth, etc.). For instance, creating a standard guide to treating LGBTQ+ adolescents has been suggested as a way to eliminate stigma in the healthcare setting and better equip individuals providing care to LGBTQ+ youth [76]. Similarly, future SUD treatment frameworks must directly take into account the specific needs of youth belonging to ethnic minorities, given the association between trauma, particularly racial trauma, and worse health outcomes [77,78,79,80,81]. SUDs are also one of the most prevalent disorders within the juvenile justice system, and the prevention, management, and treatment of SUD among youth involved in the criminal justice system should be considered in those settings [82,83]. Moreover, country-specific adaptations due to differing cultures, legislation, and philosophies of care must be considered. Although international guidelines provide clinicians with evidence in a more usable form, customizing standard practices for local use is an approach that has the potential to enhance the applicability of and adherence to measures, ultimately improving patient care. In designing the execution strategy, frontline providers and clinicians should be invited to take part in stakeholder meetings and be included in reshaping care for youth with substance use and concurrent disorders. Youth and their support systems must also be essential collaborators. Engaging them is a critical step in making services that are attractive and exciting to youth, thereby increasing their engagement with, and retention in, such services [44,57,84]. The consensus statements can inform the development of guidance documents and best practice guidelines, which should be established with input from youth themselves and their families. Further mixed-method research should engage all relevant stakeholders in the community and combine data from youth, parents, community leaders, teachers, etc., which can offer a multifaceted perspective on substance use treatment experiences and help determine the elements of prevention, management, and treatment that are effective for younger patients and their support network [56,84,85]. An international platform or committee could be created for further consultation and guidance on this topic, similar to the International Collaboration on ADHD and Substance Abuse (ICASA) [86]. Furthermore, clinical research capacity in this area is needed while recognizing the lack of well-conducted trials for this demographic, which contributes to the lack of evidence to guide clinical care [21,24,25]. A transdisciplinary collaboration across sectors is vital, especially between all members of the youth’s medical team, including paediatricians, primary care providers, psychiatrists as well as allied health members.

### Limitations

Firstly, although bias is always a possibility when using consensus techniques, the modified Delphi process is designed to minimize the influence of group dynamics or single individuals on the outcome. This bias was further minimized by using a structured process and by both open and anonymous feedback via multiple formats (e.g., group discussion, online qualitative surveys, email, and individual feedback). Secondly, one potential weakness is the possibility of bias in the experts recruited to take part in this consensus-process. As mentioned in the Methods, we tried to address this by using a number of factors when selecting participants to invite. Moreover, we recruited participants with representation mostly from Europe, Australasia, and North America, which immediately implies that the consensus reached is a product of a broad European, Australasian, and North American perspective, and certainly a product of the particular group of individuals who took part. There was a lack of representation from Asian, South American, and African countries, which may result in a bias of opinions towards consensus that may favour individualism and autonomy—values more prevalent in the countries represented. Thirdly, with regards to the small sample size, the participation of 31 experts is not out of the ordinary when compared to previously published consensus statements [49,50,51]. Though having more group members can increase the reliability of group judgement, large groups may cause coordination problems while also having subtle effects on decision-making. It has been hypothesized that below about six participants, reliability will decline quite rapidly, while above about 12, improvements in reliability will be subject to diminishing returns [51].

## 5. Conclusions

Internationally, youth mental health care and especially support for youth with high-risk substance use is substandard. A lack of research, evidence, resources, and clinician training in this field is well documented. Improvements in prevention, treatment, and management of high-risk substance use among youth must be made, and this international consensus statement is a step towards this. Much work is needed; without systemic changes, fatalities among youth will continue to rise.

## Figures and Tables

**Table 1:** Characteristics of panelists (*N* = 31).

**Country**	***n* (%)**
Australia	2 (6.5%)
Austria	5 (16.1%)
Canada	6 (19.4%)
Egypt	1 (3.2%)
Germany	3 (9.7%)
Italy	1 (3.2%)
Netherlands	2 (6.5%)
New Zealand	2 (6.5%)
Switzerland	6 (19.4%)
United States	3 (9.7)
**Profession**	***n* (%)**
Psychiatrist	21 (67.7%)
Addiction psychiatry specialist	13
Child and adolescent psychiatry specialist	4
Dual specialist in addiction psychiatry and child and adolescent psychiatry	5
Pediatrician	4 (12.9%)
Adolescent medicine specialist	2
Dual specialist in adolescent medicine and addiction medicine	1
Dual specialist in developmental-behavioural pediatrics and addiction medicine	1
Psychologist	3 (9.7%)
Youth addiction specialist	1
Addiction medicine specialist	1
Mental health and addiction specialist	1
General Practitioner	2 (6.5%)
Addiction medicine specialist	1
Psychosomatic medicine and psychotherapy specialist	1
Emergency Medicine Physician	1 (3.2%)

Legend: Country and profession of panelists.

**Table 2:** Sixty consensus statements on the prevention, treatment, and management of high-risk substance use among youth.

**A. Clinical Risks/Concurrent Conditions**	
1	Screening for mental health problems, substance use problems, and related risks in primary care and other clinical settings is recommended for all youth.
2	It is recommended that screening be performed preferably with the adolescent alone (without the parent(s)/caretaker being present), and that discussions surrounding mental challenges, substance use, and related risks to be performed in an open and non-judgmental way.
3	It is recommended that youth who are known to be at risk of SUD due to mental health problems or a family history of SUD and mental health receive targeted prevention efforts and more frequent screening.
4	It is recommended that other critical domains be assessed among youth with high-risk substance use when deemed appropriate by primary care providers. These can include psychological distress, family history and family functioning, peer group and social functioning, high-risk behaviors, physical health, housing and financial situation, employment and academic capacity, coping strategies, and resilience.
5	It is recommended that trauma be carefully and methodically assessed among youth with high-risk substance use by providers that the youth can trust and feel comfortable sharing with.
6	Among youth with high-risk substance use, it is recommended that psychiatric assessments for mental health and substance use disorders be performed routinely using clinical guidelines for screening and assessment of mental health.
7	Among youth with high-risk substance use, it is recommended that physical health assessments for frequently occurring physical conditions, such as infectious complications be performed routinely using clinical guidelines for screening and assessment of physical health.
8	Among youth with high-risk substance use presenting with their first episode of psychosis, it is important to rule out transient causes of psychosis such as substance use or medical ailments. Youth must also be assessed for primary psychotic disorders.
9	Youth with high-risk substance use presenting with severe infections such as Hepatitis C and HIV must be offered treatment according to guidelines and be provided with the best guidance to make an informed decision.
10	It is recommended that protocols be in place for any youth who experiences serious, life-threatening outcomes such as overdose, consecutive binge drinking episodes, or strong suicidal ideation.
11	Protocols for youth who experience serious life-threatening outcomes should recommend that youth be provided with immediate access to counselling, case management, appropriate pharmacotherapy treatment, and the encouragement to notify and involve social support systems.
**B. Target populations**	
1	Services must be tailored to the developmental age of the individual and be substance-specific, severity-specific, and risk-specific.
2	Having parents with SUD is one of the most prominent risk factors for youth to develop SUD in adolescence and/or early adulthood; prevention and early identification are paramount for youth whose parents suffer from SUD.
3	It is important that parents with SUD be supported in maintaining guardianship and provided with parenting guidance when appropriate; treatment should work towards stabilizing the family as a unit.
4	For youth whose parents suffer from SUD, special attention should be paid to the youth’s experiences and their relationships with their family members.
5	It is recommended that substance use and mental health care organizations routinely ask parents with SUD about family functionality to ensure the needs of the youth are being addressed. Protocols should be in place to notify specialized organizations and appropriate government ministries if there is suspicion of child abuse or family violence.
6	It is recommended that youth with high-risk substance use and living in marginalized environments be provided with supportive housing options in cooperation with engaged institutions (youth welfare services, treatment services, harm reduction services, etc.).
7	It is recommended that young females of childbearing age who are at risk of becoming pregnant whilst taking substances be provided with options for contraception with counselling and sexual health assessments as appropriate.
8	It is recommended that young females using substances who are expectant mothers be provided with access to supports that specifically address their needs including prenatal and postnatal care services, health education, and consulting services, as well as family planning services.
9	It is recommended that young females using substances who are expectant mothers be provided with support to help develop their capacity as caregivers and should be connected to services to address risks and encourage parenting. Foster care, adoption, and termination of pregnancy can also be options if desired by the expectant mother, and resources for each option should be available.
10	It is recommended that youth with concurrent conditions be specifically engaged with multidisciplinary teams specializing in dual diagnosis among youth.
11	It is recommended that youth with concurrent conditions be specifically offered psychoeducational activities to improve awareness about the triggering effects of substances and the worsening of psychiatric conditions.
12	It is recommended that youth with opioid use or regular stimulant use be provided with age-appropriate counseling, case management, family therapy, and pharmacotherapy, ideally all through the same treatment program for integrated care. Peer support, harm reduction services, selected preventive interventions, and health education should also be provided.
**C. Intervention goals**	
1	The objectives of treating youth with high-risk substance use are to reduce harm and mortality, prevent interference in adolescent development and substance-related impairment, and promote resilience and positive youth development.
2	SUD treatment should be goal-oriented, tailored to each individual, and provided in partnership with youth and others collaborating in their care.
3	It is recommended that all evidence-based interventions be available and used according to the needs and preferences of the patient in collaboration with the care team for maximal engagement.
4	Relapse is part of the symptomatology. Youth must be enabled to recognize what to do when the risk of relapse is high, or when a slip has occurred. Motivation interviewing, skills building, and mitigating risks of substance use are recommended to help attain goals.
5	It is important to discuss overdose in an open and direct way within a harm reduction framework. Youth who (intend to) use drugs should have access to a spectrum of youth-friendly harm reduction services and be encouraged to use them.
6	It is important to provide Naloxone/Narcan and education to the entire community surrounding a youth with high-risk substance use, including their family and friends.
7	It is recommended to warn youth prescribed opioids for pain management about the risks of substance misuse/overdose and be assessed frequently for step-down to appropriate medications, as directed by specific guidelines for clinicians.
8	If necessary, youth prescribed opioids for pain management and at high risk of prescription opioid misuse and/or opioid use disorder should be given Naloxone/Narcan and offered resources for prevention and treatment.
9	It is important for psychoeducation and risk management to be accessible in schools, in health systems, in vocational activities, in mainstream media, through online interventions, and in open discussions with the caring adults in their lives.
10	It is important for standard school-based programs to be provided as part of the curriculum to reduce barriers, increase primary prevention, and target early intervention for youth who are at risk.
11	It is important for all youth to have access to online tools for risk assessment and monitoring, which can provide them with the opportunity for personalised feedback, tailored information, and harm-reduction advice rapidly, while also being anonymous if desired.
**D. Evidence-based intervention strategies**	
1	It is recommended that psychosocial evidence-based intervention strategies include family therapy, motivational interviewing, counselling, cognitive behavioral therapy, and integrated treatment of concurrent disorders, and peer support should be offered to all youth with SUD.
2	For opioid use disorder, evidence-based medication treatments, including opioid agonist treatment (OAT), are recommended as the first-line intervention.
3	It is important that the range of medications for opioid use disorder (including buprenorphine, methadone, extended-release naltrexone, slow-release morphine, etc.) be available to youth and that medication choice be prioritized based on the preference and needs of the patient.
4	For high-risk cannabis use, behavioral interventions such as motivational interviewing, cognitive behavioral therapy, and family therapy are recommended.
5	For stimulant dependence, behavioral interventions such as contingency management, motivational interviewing, cognitive behavioral therapy, and family therapy are recommended. Medication may be required to manage problematic symptoms, particularly in stimulant withdrawal.
6	For benzodiazepine dependence, it is recommended that pharmacotherapy involving the gradual tapering of benzodiazepines be considered as first choice treatment if there is a chance of withdrawal. Regardless, behavioral interventions, such as contingency management, cognitive behavioral therapy, and family therapy, are also recommended along with symptomatic treatment.
7	As most new and established medications have not been systematically evaluated in young people, decisions about their use should be taken with reference to the evidence-base in adults or as evidence emerges in youth and include a collaborative risk-benefit analysis.
8	It is recommended to assess youth for informed decision-making capacity and engaged in appropriate assent or consent processes when choosing the appropriate treatment options. Parents/caretakers should be involved to support decision-making and treatment when appropriate.
9	Text message reminders, contingency management, case management, community support, and motivational interviewing are also recommended to enhance adherence and retention.
10	Educating the public, addressing the stigma, easing transition between the services, and training providers are all paramount in increasing overall access to OAT.
11	Inpatient rehabilitation should be considered for youth with high-risk substance use if this is the preference of the youth, if the social environment is toxic, if the housing situation is very unstable, if there is a long history of unsuccessful treatment attempts, or if there are severe negative medical, social, and psychological consequences to any other option.
12	Treatment approaches can be made developmentally appropriate by using language that is accessible to youth, by focusing on their goals, and by individualizing their treatment trajectories to meet their specific needs through youth/provider joint decision-making.
13	It is recommended to involve the parents/caretakers in the treatment process. Even in dysfunctional families or in the case of divorce, parents/caretakers remain a critical resource for recovery. Treatment or consultation can happen in different ways, from direct family sessions to parallel consultations. However, limited or no parental involvement should not be a barrier to treatment for youth.
14	Family involvement can be counterproductive in certain situations, such as significant family conflict, abusive relationships, violence, and estrangement. It is important for youth protection to be prioritized.
15	Youth should guide who is involved in their treatment, and this should be respected. It is critical to encourage effective autonomy while balancing that against the capacity for effective health-directed decision making. Disclosure to others collaborating in their care should be performed with careful consideration of the risks of mistrust and disengagement. However, if youth or others are at risk of significant harm, breach of confidentiality needs to be considered.
16	If the parents/caretakers do not support treatment initially (often due to a misunderstanding or preconceived notion), their involvement should continue to be encouraged via education, enhancing trust, and relieving concerns. It is important that interventional strategies work with parents/caretakers and youth in parallel, until they agree to have common sessions.
**E. Appropriate treatment settings and expertise**	
1	Peer support and case managers are quintessential to all treatment settings, with seamless transitioning and hand-off between all treatment settings.
2	It is important for emergency department and intensive care admissions to act as a youth-friendly touchpoint and gateway for screening, brief intervention, and referral to treatment.
3	It is recommended that youth admitted to emergency departments and intensive care units be immediately linked to case-managers, offered a private space for visits from caregivers, referred to substance use treatment, and be connected to therapy depending on their preferences.
4	All services, including outreach and harm reduction, must be low-threshold, youth-friendly, and stigma-free. They have to be interesting and safe to youth.
5	All services must have seamless transitions and referrals to treatment centers for youth interested.
6	Easy transportation to and from services, flexible hours that work for youth, and the possibility of online interventions (texting, social media, apps) are recommended.
7	Professionals must be empowered and feel comfortable dealing with high-risk substance use among youth, no matter their specialization. The healthcare system as a whole must be better trained in dealing with high-risk substance use among youth.
8	It is important to provide physicians involved in the care of youth with specialty-appropriate education and training for proper referral or management of high-risk substance use among youth.
9	There is an urgent need for more clinical research, such as randomized controlled trials and high-quality observational studies, which focuses on improving models of care for high-risk substance use in adolescents and young adults. Explicit clinical research on high-risk substance use among adolescents and young adults needs to become a priority.
10	Critical data about serious adverse events among youth who use substances, including non-fatal and fatal overdose events, as well as about treatment capacity (including the number of young patients dispensed OAT and the number of youth OAT prescribers), must be collected, analyzed, and reported in a timely fashion.

Legend: Agreement with the statement was rated on a 5-point scale (1 = strongly disagree, 2 = somewhat disagree, 3 = neutral, 4 = somewhat agree, and 5 = strongly agree). In each round, consensus for each statement was reached if less than two experts provided a score of 2 or lower. SUD: substance use disorder; OAT: opioid agonist treatment; HIV: human immunodeficiency viruses.

## Data Availability

All data are presented in the paper, but raw data can be made available by reasonable request to the corresponding author.

## Supplementary Materials

The following are available online at https://www.mdpi.com/article/10.3390/medicina58040539/s1, Supplementary Material S1: Prevention & treatment of high-risk substance use among youth-open-ended questionnaire.
