# Supplementary material for: Towards an International Consensus on the Prevention, Treatment, and Management of High-Risk Substance Use and Overdose among Youth
Source: Medicina (Kaunas). 2022 Apr 13;58(4):539. doi: 10.3390/medicina58040539 (PMC9028633; doi:10.3390/medicina58040539)
Supplement: Supplementary file 1 [file medicina-58-00539-s001.zip › medicina-1645964-supplementary.pdf]

PREVENTION & TREATMENT OF HIGH-RISK SUBSTANCE USE AMONG YOUTH - OPEN-ENDED QUESTIONNAIRE

A. OUTLINING CLINICAL RISKS/CONCURRENT CONDITIONS

1) What are the primary substances for which youth enter treatment in your city, country?

2) What substances are associated with the most severe adverse consequences in your country?

3)How has the overdose crisis effected youth in your country?

4) Do you think that the clinical assessment and the focus for substance use should include adolescents (12-17 years) and young adults or transitioning youth (18-25)? Should they be addressed and evaluated in a common target age frame?

5) What is your experience with high-risk substance use among youth and give examples of the most concerning developments in your country?

6) Which assessment domains and psychometric tools are critical for the understanding and monitoring of high-risk substance use among youth, defining high-risk as the likelihood of serious adverse events (overdose, self-harm, binge drinking)?

In prevention:

In treatment:

7) Which domains and psychometric tools can be used for “online risk assessment and monitoring”? Are you aware of existing developments or solutions?

In prevention:

In treatment:

8) Should the assessment of user's engagement and satisfaction be part of routine assessment and when should it happen?

9) How should the substance use best be documented and monitored? Are there youth specific categories or items?

10) Below are critical domains beyond immediate substance use. How should they be included and addressed? Please also suggest additional critical domains that are not listed.

- Family
- Social/relational/interpersonal
- Early trauma
- Mental health risks
- Cognitive capacity and academic/vocational achievement
- High-risk behaviors (unprotected sexual activity, drinking and driving), etc.
- Others:

11) Below are severe adverse events related to risky substance use. How should they be addressed? Please suggest additional severe adverse events and how they should be addressed.

- Overdose
- Binge drinking
- Injection
- Emergency room visits
- Suicidal ideations and attempts
- Crisis/acute clinical admission
- Psychosis
- Others:

B. DETERMINING TARGET POPULATIONS

1) Given their unique issues, adolescents with substance use disorder benefit from services designed specifically for them. Among youth, there are different age categories, levels of substance use and risk-taking, reasons for substance use, and preventative and therapeutic needs. How would identifying specific subpopulations aid in the prevention and treatment of adolescents with SUD?

2) Consider and comment the following cluster:

- Adolescents whose parents suffer from substance abuse

- Adolescents with exploratory use

- Adolescents with high-risk exploratory use of non-opiates (binge drinking, intoxication in public, chain smoking, stimulants etc.) and or living in a marginalized environment (foster care, homelessness, forensic etc.)

- Adolescents with severe concurrent conditions (suicide attempts, violence, severe trauma, psychosis)

- Adolescents with exploratory opioid use or regular crack-cocaine use

- Adolescents injecting opioids or other psychotropic substances

Are there any specific target populations that have not been mentioned?

C. DEFINING INTERVENTION GOALS

1) What should be the main goals of treatment for youth with SUD which should always be included in the treatment planning?

2) When should harm reduction be an appropriate treatment goal for youth?

3) How should harm reduction services be organized? Should they always be youth specific? Please give examples.

4) How should overdose prevention be integrated into the system?

5) In your perspective, what are the 3 main risk factors for overdose among youth?

1.
2.
3.

6) How should youth be taught about their risks and how to manage them?

7) When and how should abstinence vs. controlled use be addressed in the treatment trajectory of youth?

8) Should screening and risk assessment of the general child and adolescent population be routinely integrated into youth mental health care? How about among youth with a family history of SUD or mental health? Should screening happen for all users following a certain age cut-off as an opportunity to assess for use, provide psychoeducation and engage in discussion in future? If so, what age range or cut-off be best?

9) Should school-based programs be implemented in general? If so, are you aware of any effective evidence-based programs that should be standardized?

10) Do you think online resources should be used to support these strategies? Are you aware of existing models?

D. RECOGNIZING EVIDENCE-BASED INTERVENTION STRATEGIES in the treatment of youth using psychotropic substances to prevent severe adverse events and support the recovery.

1) Consider the following: youth who do not meet criteria for SUD or have at risk use should be offered age-appropriate advice and/or an extended brief intervention to discourage further use. These interventions should be youth adapted and engaging. How can that be achieved? Consider the following strategies:

- Harm reduction
- Overdose prevention
- Risk management
- Motivational enhancement
- Behavioral management

2) Please rate these interventions on a 5-point scale from the following list in relation to their significance and importance as a core set of treatment for youth, 0 being "not at all important" and 5 being "extremely important".

- Safe injection sites:
- Harm reduction programs:
- Drug testing programs:
- Contingency Management:
- Detoxification and withdrawal management:
- Pharmacologic interventions:
- Long term abstinence-based inpatient rehabilitation:
- Family therapy (e.g., MST, CRA, CRAFT):
- Psychotherapy/Counselling (e.g., CBT, CM, MI, etc.):
- Integrated treatment of comorbid disorders:
- Mental health care:
- Physical health care:
- Psychosocial/peer support:

3) Among the pharmacological interventions primarily aimed at treating SUD, which do you consider to be the first line for youth and why? Consider the following substances being used:

- Nicotine:
- Alcohol:
- Opioids:
- Cannabis:
- Stimulants:
- Others:

Are there useful strategies not following guidelines and if so which?

4) Among adults, opioid agonist treatment (OAT) is commonly done with Methadone, Suboxone (buprenorphine/naloxone), Kadian (slow-release oral morphine), etc. In your experience, are any safer and more effective for youth? Should any of these opioid agonist medications be especially be recommended or emphasized in the treatment for youth?

5) In your country, what is the prevalence of youth with high-risk opioid use/OUD in OAT? Please bold from one of the options below and provide additional descriptions if possible.

OAT is the normal available treatment option
OAT is only used as exception
OAT is not recommended and not used

Additional description of the coverage of OAT for youth in your country:

6) In your country, are the needs of youth being reached? If not, please outline the 3 most important steps to achieve this.

7) How can the number of youths retained in OAT be improved?

8) When should long term abstinence-based inpatient rehabilitation be considered?

9) Among the psychotherapeutic interventions, which do you consider to be the first line for youth and why?

10) What are the different evidence-based psychotherapeutic interventions that you would recommend using for youth?

11) How do you make treatment approaches/plans for youth developmentally appropriate?

12) Parental/family involvement in the treatment of youth with SUD is complicated. Adolescents may still live at home in which case parental involvement would be relevant. It might be mandatory to involve parents in the treatment of minors in some countries. Regardless, parental involvement should not be barrier to treatment. When is it appropriate for a young person's parent or carer to be involved in the treatment with youth? Conversely, when is it less appropriate/helpful to involve the parents or caregivers?

E. IDENTIFYING APPROPRIATE TREATMENT SETTINGS AND EXPERTISE

1) What should define the core of treatment and recovery for youth?

2) How could youth be best supported in the following treatment settings:

2a) Harm reduction facilities

2b) Low-threshold facilities (such shelters, meal services, etc.):

2c) Emergency rooms (in case of OD, binge-drinking, etc.):

2d) Acute care inpatient:

2e) Day clinics:

2f) Outpatient:

2g) Outreach:

2h) Intensive Care Medicine:

2i) Long-term rehab:

3) What expertise or specialty should be principal to the treatment and recovery of youth? Consider the following:

- Pediatrics
- Primary care
- Adolescent Psychiatry
- Adult Psychiatry
- Addiction Psychiatry
- Adolescent Medicine
- Other professions (e.g., social workers, etc.)
- Others not mentioned:

4) How should medications be best provided in treating youth with SUD?

5) How should be the relationship between substance use specialists and other primary care professionals in the treatment of youth mental health problems?
